# Unmet Hearing Aid Need Linked to Lower Community Engagement Satisfaction in Vulnerable Older Adults

**DOI:** 10.1093/geroni/igaf122.3413

**Published:** 2025-12-31

**Authors:** Dana Urbanski, Jack Wolf, Romil Parikh, Benjamin Langworthy, Eric Jutkowitz, Tetyana Shippee

**Affiliations:** Indiana University Bloomington, Bloomington, Indiana, United States; University of Minnesota School of Public Health, Minneapolis, Minnesota, United States; University of Minnesota School of Public Health, Minneapolis, Minnesota, United States; University of Minnesota School of Public Health, Minneapolis, Minnesota, United States; University of Minnesota, Minneapolis, Minnesota, United States; University of Minnesota, Minneapolis, Minnesota, United States

## Abstract

Hearing loss is associated with dementia, possibly by driving social isolation that contributes to cognitive decline. Hearing aids can improve social engagement among older adults with hearing loss, yet only one-third of those who could benefit from hearing aids use them—often due to barriers such as cost, access, and lack of social support. Consequently, many vulnerable older adults report unmet hearing aid need; however, little research has examined social/community engagement in this population, which may be amenable to treatment but has not received hearing aids. This study examined a single-item measure of community engagement satisfaction among older adults receiving publicly funded home- and community-based services (HCBS; an indicator of social and financial vulnerability) stratified by self-reported hearing aid need. Using data from the 2017-2019 National Core Indicators-Aging and Disability Adult Consumer Surveys, the sample included 10,861 HCBS users aged ≥65 years, 14% of whom reported unmet hearing aid need. Unadjusted satisfaction rates were lower for those who reported unmet need (38.8%) compared to those who reported not needing hearing aids (49.4%) or already using hearing aids (52.3%). Adjusting for covariates, a Poisson model with robust standard errors pooled across multiple imputations revealed that HCBS users reporting unmet need were still 12% (95% CI, 5%-19%) and 17% (95% CI, 11%-24%) less likely to report satisfaction with their community engagement compared to those who reported not needing or already using hearing aids, respectively. Addressing unmet hearing aid need among vulnerable older adults may promote social and community engagement.

